# Autonomic Regulation of the Goldfish Intact Heart

**DOI:** 10.3389/fphys.2022.793305

**Published:** 2022-02-09

**Authors:** Maedeh Bazmi, Ariel L. Escobar

**Affiliations:** ^1^Quantitative Systems Biology Program, School of Natural Sciences, University of California, Merced, Merced, CA, United States; ^2^Department of Bioengineering, School of Engineering, University of California, Merced, Merced, CA, United States

**Keywords:** electrocardiogram, local field fluorescence microscopy, intracellular microelectrodes, sympathetic regulation, parasympathetic regulation

## Abstract

Autonomic regulation plays a central role in cardiac contractility and excitability in numerous vertebrate species. However, the role of autonomic regulation is less understood in fish physiology. Here, we used Goldfish as a model to explore the role of autonomic regulation. A transmural electrocardiogram recording showed perfusion of the Goldfish heart with isoproterenol increased the spontaneous heart rate, while perfusion with carbamylcholine decreased the spontaneous heart rate. Cardiac action potentials obtained *via* sharp microelectrodes exhibited the same modifications of the spontaneous heart rate in response to isoproterenol and carbamylcholine. Interestingly, the duration of the cardiac action potentials lengthened in the presence of both isoproterenol and carbamylcholine. To evaluate cardiac contractility, the Goldfish heart was perfused with the Ca^2+^ indicator Rhod-2 and ventricular epicardial Ca^2+^ transients were measured using Pulsed Local Field Fluorescence Microscopy. Following isoproterenol perfusion, the amplitude of the Ca^2+^ transient significantly increased, the half duration of the Ca^2+^ transient shortened, and there was an observable increase in the velocity of the rise time and fall time of the Ca^2+^ transient, all of which are compatible with the shortening of the action potential induced by isoproterenol perfusion. On the other hand, carbamylcholine perfusion significantly reduced the amplitude of the Ca^2+^ transient and increased the half duration of the Ca^2+^ transient. These results are interesting because the effect of carbamylcholine is opposite to what happens in classically used models, such as mouse hearts, and the autonomic regulation of the Goldfish heart is strikingly similar to what has been observed in larger mammalian models resembling humans.

## Introduction

In nearly all vertebrate species, direct input from the autonomic nervous system tightly controls cardiac contractility and excitability (Lee and Shideman, 1959; Katz, 1967; Lindemann and Watanabe, 1985; Cohn, 1989; Henning, 1992). Although there is an abundant amount of research on the autonomic control of cardiac contractility and excitability in numerous mammalian species, the characterization of pathophysiological mechanisms is still difficult to obtain for humans specifically. This is in part due to humans having strikingly dissimilar action potential (AP) characteristics and electrocardiographic morphology in comparison with commonly used animal models such as mice, rats, and rabbits (Nakamura et al., 2002; Tsai et al., 2011; Bazmi and Escobar, 2020). Fish, on the other hand, are the largest and most diverse group of vertebrates, and as such, their autonomic nervous system regulation can often deviate from the classical vertebrate models used to study autonomic control of cardiac contractility and excitability. The most drastic difference in autonomic system regulation can be observed when comparing the hagfish, which have no known autonomic nervous system control, to the teleost, which exhibit fully functional autonomic regulation in cardiac function (Sandblom and Axelsson, 2011). Nevertheless, if a fish species does exhibit autonomic regulation, it is likely to be similar to what has been established for many mammalian species.

In vertebrate species exhibiting full autonomic control, the autonomic nervous system functions through two closely intertwined antagonistic branches: the sympathetic branch and the parasympathetic branch. The sympathetic branch of the nervous system, referred to as the sympathetic nervous system, modulates cardiac function through the release of transmitters referred to as catecholamines (Lee and Shideman, 1959; Evans, 1986; Marks, 2013). These catecholamines bind to and stimulate 𝛽-adrenergic receptors, which in turn, increase the speed of conduction through the atrioventricular node (positive dromotropic effect), increase heart rate (positive chronotropic effect), increase contractility (positive inotropic effect), and increase the velocity of myocardial relaxation during diastole (positive lusitropic effect). Locally released catecholamines, such as norepinephrine (NE), stimulate the 𝛽-adrenergic receptors by activating adenylyl cyclase (AC; Hildebrandt et al., 1983; Brum et al., 1984) and increasing cyclic adenosine monophosphate (cAMP) levels (Osterrieder et al., 1982). Increased cAMP levels activate protein kinase A (PKA; Krebs, 1972; Hayes and Mayer, 1981) and induce the dissociation of the catalytic subunit. Levels of cAMP and thus PKA are finely regulated by cyclic nucleotide phosphodiesterases (PDEs) which degrade cAMP into 5′-AMP. Nevertheless, the catalytic subunit of PKA phosphorylates several key Ca^2+^ handling proteins such as the L-type Ca^2+^ Channel (LTCC; Collins et al., 1981; Osterrieder et al., 1982), the ryanodine receptor 2 (Suko et al., 1993; Valdivia et al., 1995), and phospholamban (PLN; Weilenmann et al., 1987). These modifications not only alter the electrical activity of the myocardium, which have positive dromotropic and chronotropic effects, but also Ca^2+^ handling dynamics in the myocardium which lead to positive inotropic and lusitropic effects (Aguilar-Sanchez et al., 2019).

The sympathetic branch of the nervous system is highly antagonized by the parasympathetic branch. Referred to as the parasympathetic nervous system, this branch modulates cardiac contractility and excitability through the local release of the transmitter acetylcholine (ACh) from postganglionic cholinergic intracardiac neurons. The ACh subsequently binds to and stimulates muscarinic (M2) receptors. Activation of M2 receptors stimulates a *G_i_* protein, which inhibits AC (Krebs, 1972). This inhibition leads to significantly lower levels of cAMP, a reduced fraction of activated PKA, and a decreased degree of phosphorylation in the key Ca^2+^ handling proteins. These modifications result in negative inotropic, chronotropic, dromotropic, and lusitropic effects, all of which are crucial in countering the sympathetic nervous system and maintaining homeostasis in the vertebrate central nervous system (Watanabe and Lindemann, 1984; Aguilar-Sanchez et al., 2019).

Although fish hearts contain a single atrium and ventricle and present a fundamentally different cardiovascular system when compared to other mammalian models, there are many developmental, structural, and functional commonalities between the two vertebrate species (Sandblom and Axelsson, 2011; Mersereau et al., 2015; Xing et al., 2017; Bazmi and Escobar, 2020). The Goldfish specifically, has remarkably similar electrical properties to humans. For example, the heart rate, AP morphology, and Ca^2+^ transient kinetics and dynamics of adult Goldfish closely parallel those of humans, even more so than mice and Zebrafish models (Bazmi and Escobar, 2020).

Previous literature suggests few fish models exhibit autonomic control in a similar manner to larger mammals; however, it is not clear how autonomically driven AP kinetics impact cardiac contractility in the fish intact heart specifically. To explore how stimulation of either autonomic nervous system branch would alter cardiac contractility and excitability, we performed experiments in which we perfused the Goldfish intact heart with either a sympathetic or parasympathetic agonist. Ventricular APs, electrocardiograms, and Ca^2+^ transients recorded from the Goldfish intact heart showed perfusion with either 100 nm isoproterenol (sympathetic agonist) or 5 μm carbamylcholine (parasympathetic agonist), was enough to stimulate the sympathetic branch or parasympathetic branch, respectively. Interestingly, our results indicate stimulation of the Goldfish autonomic nervous system by these commonly used agonists resulted in a corresponding change in cardiac dromotropism, chronotropism, inotropism, and lusitropism in a similar manner observed in humans.

## Materials and Methods

### Ethical Approval

Our animal facilities are Association for Assessment and Accreditation of Laboratory Animal Care accredited and Office of Laboratory Animal Welfare certified and fully comply with all regulations, policies, and standards that protect animal welfare. Animal use in our studies were in accordance with the National Institutes of Health Guide for the Care and Use of Laboratory Animals (NIH Publication No. 85–23, Revised 1996) and the Institutional Animal Care and Use Committee guidelines of the University of California Merced (Protocol # 2008–201). The animals were bought from Toledo Goldfish, United States.

Adult Goldfish were anesthetized by immersion in ice-cold water containing 0.16 mg ml^–1^ tricaine methanesulfonate for 2–5 min. To assure the Goldfish were completely anesthetized prior to decapitation, the tail was held with a small, curved tweezer, and gently moved. Once the Goldfish were completely anesthetized, they were decapitated, and the intact heart was removed from the chest cavity.

### Heart Cannulation and Perfusion

Goldfish hearts were dissected, and the bulbous arteriosus was cannulated onto a gauge 27 needle and perfused in a Langendorff system at a rate of 60 μl/min driven by gravity. Multiple solutions were perfused through the bulbus arteriosus with the aid of a self-designed μ-manifold. Goldfish hearts were perfused with a fish ringer solution containing: NaCl 137 mm, KCl 5.4 mm, CaCl_2_ 1.8 mm, MgCl_2_ 0.5 mm, HEPES 10 mm, and glucose 5.5 mm. The Ca^2+^ dye Rhod-2 AM was perfused into the heart with a Harvard pump for 30–45 min. The temperature of the bath containing the heart was set to 28°C. The temperature was controlled with the aid of a Peltier unit positioned at the bottom of the recording chamber and measured with a linearized semiconductor temperature sensor.

### Pharmacological Agents

The Goldfish heart was perfused with fish ringer solution containing 4 mm blebbistatin, prior to obtaining any electrophysiological recordings to suppress cardiac motion. In order to elicit a sympathetic response, the Goldfish heart was perfused with fish ringer solution containing 100 nm isoproterenol for 10 min before the start of any AP and Ca^2+^ transient recordings. To determine if the sympathetic response to isoproterenol could be reversed, the Goldfish heart was perfused with fish ringer solution for a prolonged amount of time. Indeed, the effects of isoproterenol could be completely reversed if the Goldfish heart were continuously perfused with fish ringer solution for 20 min. In contrast, to elicit a parasympathetic response the Goldfish heart was perfused with fish ringer solution containing 5 μm carbamylcholine for 10 min prior to the start of any AP and Ca^2+^ transient recording. The effects of carbamylcholine could be completely reversed after continuously perfusing the heart with fish ringer solution for 60 min. Recordings obtained prior to perfusion with isoproterenol or carbamylcholine were considered as control, and recordings obtained following isoproterenol or carbamylcholine perfusion were considered as experimental.

### Experimental Setup

#### Optical Measurements

Ca^2+^ transients were recorded (*N* = 8 hearts) using Pulsed Local Field Fluorescence Microscopy (PLFFM; Mejía-Alvarez et al., 2003; Escobar et al., 2004, 2006; Valverde et al., 2006, 2010; Kornyeyev et al., 2010; Mattiazzi et al., 2015; Aguilar-Sanchez et al., 2017). The PLFFM technique assessed physiological parameters by exciting exogenous probes present in the tissue and detecting the light emitted by these fluorescent indicators. The excitation (532 nm Yag laser) and emitted light propagated through a multimode fiber optic (200 mm diameter, 0.67 NA) placed on the surface of the intact heart. The emitted light then traveled back through the multimode fiber, dichroic mirrors, and filters (610 nm) and was focused on an avalanche photodiode (Perkin Elmer, United States) with the aid of a microscope objective. The signal was digitized by an A/D converter (NI, United States) and acquired by a PC. The fluorescent indicator utilized to obtain Ca^2+^ transients in this study was Rhod-2 AM. Often referred to as a “Ca^2+^ indicator dye,” Rhod-2 AM (50 μg) was prepared with 20 μl of 20% pluronic in 1 ml fish ringer solution.

#### Electrophysiological Measurements

Epicardial electrical recordings of the APs (*N* = 4 hearts) were obtained using sharp glass microelectrodes filled with 3 M KCl connected to a high input impedance differential amplifier (WPI, United States). Glass microelectrodes were fabricated with a micropipette puller (Sutter Instrument Co., United States) and had a resistance of 10–20 MΩ (Ferreiro et al., 2012; López Alarcón et al., 2019). Data were recorded with an acquisition system from National Instruments in conjunction with additional software built in our lab. All fluorescence and membrane potential recordings were obtained from the Goldfish ventricular epicardium. Goldfish hearts were continuously paced at 1 Hz with the aid of two acupuncture needles placed in the apex of the ventricle in the presence and absence of the pharmacological agents. However, the hearts were not paced in experiments assessing changes in the spontaneous heart rate.

#### Whole Heart Electrocardiographic Measurements

Transmural electrocardiographic recordings (*N* = 6 hearts) were performed by placing one Ag–AgCl micropellet inside the left ventricle and a second pellet outside the left ventricle (Kornyeyev et al., 2010; López Alarcón et al., 2019). Signals were amplified by a custom-made DC-coupled instrumentation amplifier and were digitally sampled identically to the AP recordings.

### Statistical Analysis

In whole heart experiments, there are two main causes of variance. First, no two animals have entirely identical hearts, regardless of the species. Second, although we are measuring Ca^2+^ transients and APs in the same region (the mid-region of the left ventricle) of the heart, it is impossible to perform the recordings in the same precise location between different hearts. Thus, the data are presented as the measured times with their standard error (SEM). To assess electrical changes, AP traces were first normalized then evaluated at their respective repolarization times. Specifically, the time it takes for the AP to reach 30, 50%, or 90% repolarization, referred to here as APD30, APD50, or APD90, respectively.

Each wave of the electrocardiogram (QRS complex, T wave, and J wave) was measured using its corresponding half duration. The recorded Ca^2+^ transients were normalized between zero (minimum fluorescence) and one (maximum fluorescence) in order to evaluate the kinetics of the recorded Ca^2+^ transients. The kinetic parameters of the Ca^2+^ transients evaluated were the rise time (time for the Ca^2+^ transient to rise from, 10 to 90% of its maximum amplitude), half duration (duration of the Ca^2+^ transient at 50% of the maximum amplitude), and fall time (time for the Ca^2+^ transient to fall from 90 to 10% of the maximum amplitude). The AP parameters and Ca^2+^ transient kinetics obtained for the control and the experimental groups were evaluated and normalized to their respective control values for each heart used. After this normalization, data were compiled, and statistical analysis was performed.

The data are presented as multiple measurements (*n*; dot cloud) recorded for different measurements (*n*) on different hearts (*N*) with the mean ± SEM (solid lines). To determine if the agonists produced a significant effect, the distribution of the data before and after administration was compared using a two-sample Kolmogorov–Smirnov test (OriginPro, 2019). The difference was significant if the value of *p* < 0.01.

## Results

### Sympathetic Regulation of Action Potentials and Heart Rate

**Figure 1:**
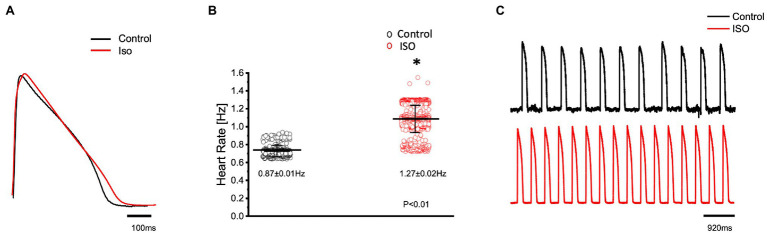
Goldfish ventricular action potential and spontaneous heart rate recordings before (black) and after perfusion with 100 nm isoproterenol (red). Perfusion of the Goldfish intact heart with 100 nm isoproterenol altered the action potential morphology **(A)** and had a positive chronotropic effect, significantly increasing the heart rate (**B**; from 0.87 ± 0.01 Hz to 1.27 ± 0.02 Hz, *p* < 0.01, *n* = 160 for the control, *n* = 263 for ISO, *N* = 4). The positive chronotropic effect following isoproterenol perfusion is also reflected in spontaneous AP recordings from the left ventricle **(C)**. ^*^Denotes a significant difference between the two distributions. The data are presented as multiple measurements (n; dot cloud) recorded for different measurements (*n*) on different hearts (*N*) with the mean ± SEM (solid lines).

**Figure 2:**
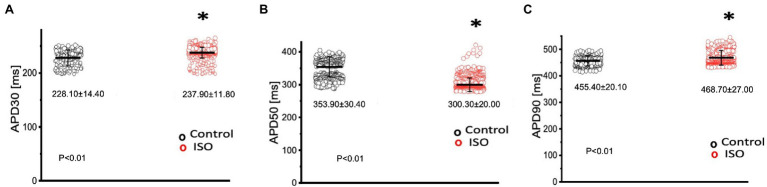
Kinetic parameters of the Goldfish ventricular action potential before (black) and after (red) perfusion with 100 nm isoproterenol. Following isoproterenol perfusion, APD30s significantly increased from 228.10 ± 14.40 ms to 237.90 ± 11.80 ms (*p* < 0.01, *n* = 113 for the control, *n* = 113 for ISO, *N* = 4; **A**), APD50 decreased from 353.90 ± 30.40 ms to 300.30 ± 20.00 ms (*p* < 0.01, *n* = 143 for the control, *n* = 316 for ISO, *N* = 4; **B**), and APD90 significantly increased from 455.40 ± 20.10 ms to 468.70 ± 27.00 ms (*p* < 0.01, *n* = 95 for the control, *n* = 256 for ISO, *N* = 4; **C**). ^*^Denotes a significant difference between the two distributions. The data are presented as multiple measurements (n; dot cloud) recorded for different measurements (*n*) on different hearts (*N*) with the mean ± SEM (solid lines).

In order to elicit a sympathetic response and assess the 𝛽-adrenergic regulation of the Goldfish heart, we first perfused the heart with 100 nm isoproterenol. Goldfish ventricular chronotropic properties were examined *via* AP recordings and spontaneous heart rate recordings (Figure 1). Perfusion of the Goldfish intact heart with 100 nm isoproterenol altered the AP morphology (Figure 1A) and had a positive chronotropic effect, significantly increasing the heart rate by 46% (Figures 1B,C; from 0.87 ± 0.01 Hz to 1.27 ± 0.02 Hz). Interestingly, all kinetic parameters of the AP significantly changed following isoproterenol perfusion; APD30 increased from 228.10 ± 14.40 ms to 237.90 ± 11.80 ms (Figure 2A), APD50 decreased from 353.90 ± 30.40 ms to 300.30 ± 20.00 ms (Figure 2B), and APD90 increased from 455.40 ± 20.10 ms to 468.70 ± 27.00 ms (Figure 2C).

### Sympathetic Prevalence in Electrocardiographic Signals

**Figure 3:**
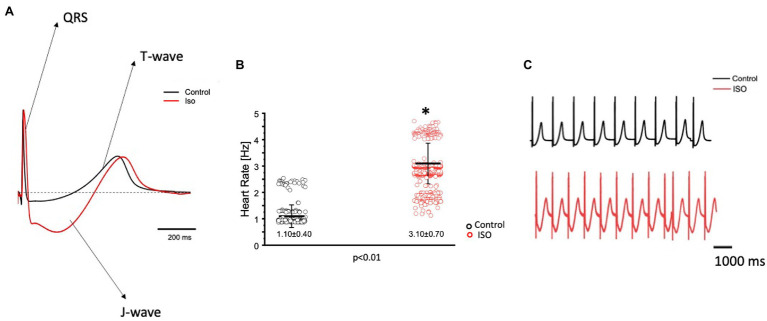
Goldfish ventricular electrocardiogram recordings before (black) and after (red) perfusion with 100 nm isoproterenol. The three main components of the Goldfish electrocardiogram are presented: QRS complex, J wave, and T wave. Application of isoproterenol altered the morphology of the Goldfish electrocardiogram **(A)** and increased the spontaneous heart rate from 1.10 ± 0.40 Hz to 3.10 ± 0.70 Hz (*p* < 0.01, *n* = 180 for the control, *n* = 412 for ISO, *N* = 6; **B**). Electrocardiogram recordings before and after isoproterenol perfusion reflect an increased heart rate in response to isoproterenol **(C)**. ^*^Denotes a significant difference between the two distributions. The data are presented as multiple measurements (n; dot cloud) recorded for different measurements (*n*) on different hearts (*N*) with the mean ± SEM (solid lines).

**Figure 4:**
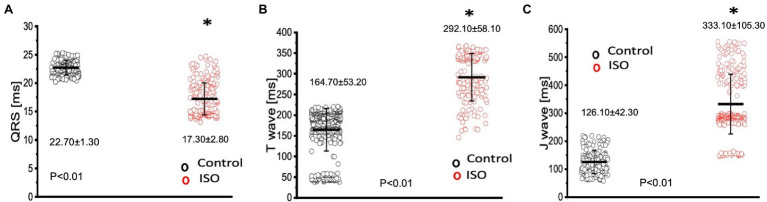
Perfusion with 100 nm isoproterenol significantly altered the time course of all three components in the Goldfish electrocardiogram. The duration of the QRS complex significantly decreased from 22.70 ± 1.30 ms to 17.30 ± 2.80 ms (*p* < 0.01, *n* = 80 for the control, *n* = 121 for ISO, *N* = 6; **A**), T wave significantly increased from 164.70 ± 53.20 ms to 292.10 ± 58.10 ms (*p* < 0.01, *n* = 110 for the control, *n* = 120 for ISO, *N* = 6; **B**), and the J wave significantly increased from 126.10 ± 42.30 ms to 333.10 ± 105.30 ms (*p* < 0.01, *n* = 74 for the control, *n* = 118 for ISO, *N* = 6; **C**). ^*^Denotes a significant difference between the two distributions. The data are presented as multiple measurements (n; dot cloud) recorded for different measurements (*n*) on different hearts (*N*) with the mean ± SEM (solid lines).

The effects of catecholamines on whole heart electrical activity were assessed through transmural electrocardiogram recordings (Figure 3). The 3 main components of the Goldfish electrocardiogram are presented in Figure 3A and consist of the QRS complex (ventricular depolarization), J wave (likely due to a voltage gradient due to the presence of a prominent AP notch in the epicardium but not the endocardium), ending with T wave (ventricular repolarization). Application of isoproterenol altered the morphology of the Goldfish electrocardiogram (Figure 3A) and increased the heart rate (Figures 3B,C; from 1.10 ± 0.40 Hz to 3.10 ± 0.70 Hz). Furthermore, application of isoproterenol also significantly altered the duration of the QRS wave, T wave, and J wave (Figure 4). The QRS complex significantly decreased from 22.70 ± 1.30 ms to 17.30 ± 2.80 ms (Figure 4A), the T wave significantly increased from 164.70 ± 53.20 ms to 292.10 ± 58.10 ms (Figure 4B), the J wave significantly increased from 126.10 ± 42.30 ms to 333.10 ± 105.30 ms (Figure 4C).

### Sympathetic Regulation of Cardiac Contractility

**Figure 5:**
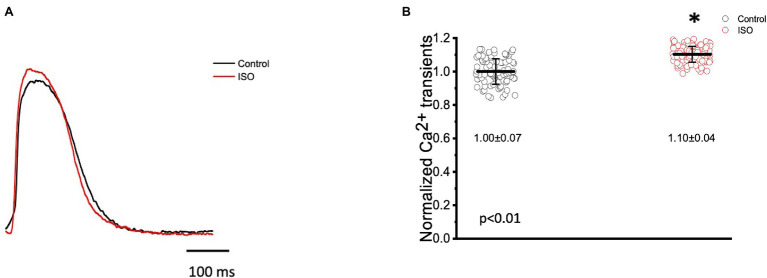
Goldfish ventricular Ca^2+^ transient recording and normalized amplitude in the absence (black) and presence (red) of 100 nm isoproterenol. Stimulation of 𝛽-adrenergic receptors altered the morphology of the Ca^2+^ transient **(A)** and significantly increased the normalized amplitude of the Ca^2+^ transient from 1.00 ± 0.07 to 1.10 ± 0.04 (*p* < 0.01, *n* = 65 for the control, *n* = 67 for ISO, *N* = 8; **B**). ^*^Denotes a significant difference between the two distributions. The data are presented as multiple measurements (n; dot cloud) recorded for different measurements (*n*) on different hearts (*N*) with the mean ± SEM (solid lines).

**Figure 6:**
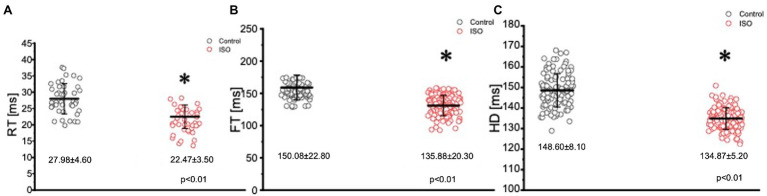
Kinetic parameters of the Goldfish ventricular Ca^2+^ transients before (black) and after (red) perfusion with 100 nm isoproterenol. Perfusion of the Goldfish heart with 100 nM isoproterenol significantly decreased the rise time (RT) of the Ca^2+^ transient from 27.98 ± 4.60 ms to 22.47 ± 3.50 ms (*p* < 0.01, *n* = 65 for the control, *n* = 96 for ISO, *N* = 8; **A**), decreased the fall time (FT) of the Ca^2+^ transient from 150.08 ± 22.80 ms to 135.88 ± 20.30 ms (*p* < 0.01, *n* = 76 for the control, *n* = 76 for ISO, *N* = 8; **B**), and significantly decreased the half duration (HD) of the Ca^2+^ transient from 148.60 ± 8.10 ms to 134.87 ± 5.20 ms (*p* < 0.01, *n* = 64 for the control, *n* = 72 for ISO, *N* = 8; **C**). ^*^Denotes a significant difference between the two distributions. The data are presented as multiple measurements (n; dot cloud) recorded for different measurements (*n*) on different hearts (*N*) with the mean ± SEM (solid lines).

In many vertebrate species, stimulation of either autonomic nervous system branch will not only alter cardiac excitability, but also cardiac contractility. In order to assess if eliciting a sympathetic response altered the inotropic and/or the lusitropic properties of the Goldfish ventricle, experiments were performed in which the amplitude and kinetics of the Ca^2+^ transient were examined in the presence and absence of 100 nm isoproterenol (Figure 5). Stimulation of 𝛽-adrenergic receptors altered the morphology of the Ca^2+^ transient (Figure 5A) and significantly increased the normalized amplitude of the Ca^2+^ transient (Figure 5B; from 1.00 ± 0.07 to 1.10 ± 0.04). To detect if isoproterenol significantly altered the kinetics of the Ca^2+^ transient, the three following parameters of the Ca^2+^ transient were assessed (Figure 6): rise time (RT), fall time (FT), and half duration (HD). A significant change in any aforementioned kinetical parameter is a reflection of a significant corresponding change in myocardial Ca^2+^ handling dynamics. Perfusion of isoproterenol significantly increased the velocity of every Ca^2+^ transient kinetic parameter in the Goldfish heart (Figures 6A–C; RT: from 27.98 ± 4.60 ms to 22.47 ± 3.50 ms, FT: from 150.08 ± 22.80 ms to 135.88 ± 20.30 ms, and HD: from 148.60 ± 8.10 ms to 134.87 ± 5.20 ms), implying perfusion with isoproterenol increased the rate of relaxation of the Goldfish myocardium during diastole, resulting in a positive lusitropic effect (Figure 6B). The presence of a 𝛽-adrenergic drive suggests the presence of a parasympathetic one, as they are the two antagonistic branches of the autonomic nervous system.

### Parasympathetic Regulation of Action Potentials and Heart Rate

**Figure 7:**
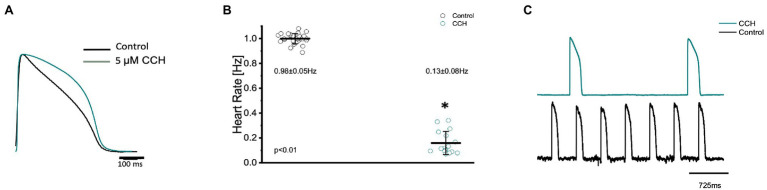
Goldfish ventricular action potential and spontaneous heart rate recordings before (black) and after perfusion with 5 μm carbamylcholine (green). Administration of carbamylcholine altered the AP morphology **(A)** and had a negative chronotropic effect, reducing the heart rate from 0.98 ± 0.05 Hz to 0.13 ± 0.08 Hz (*p* < 0.01, *n* = 29 for the control, *n* = 16 for CCH, *N* = 4; **B**). The negative chronotropic effect following carbamylcholine perfusion is also reflected in spontaneous AP recordings from the left ventricle **(C)**. ^*^Denotes a significant difference between the two distributions. The data are presented as multiple measurements (*n*; dot cloud) recorded for different measurements (*n*) on different hearts (*N*) with the mean ± SEM (solid lines).

**Figure 8:**
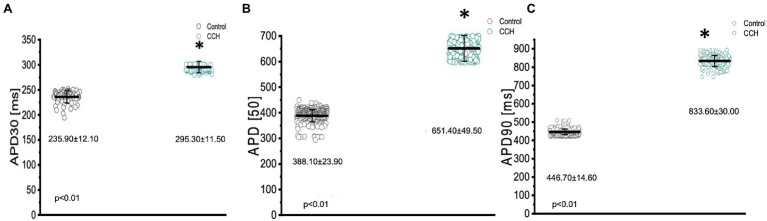
Kinetic parameters of the Goldfish ventricular action potential before (black) and after (green) perfusion with 5 μm carbamylcholine. APD30 significantly increased from 235.90 ± 12.10 ms to 295.30 ± 11.50 ms (*p* < 0.01, *n* = 80 for the control, *n* = 49 for CCH, *N* = 4; **A**), APD50 significantly increased from 388.10 ± 23.90 ms to 651.40 ± 49.50 ms (*p* < 0.01, *n* = 379 for the control, *n* = 308 for CCH. *N* = 4; **B**), and APD90 significantly increased from 446.70 ± 14.60 ms to 833.60 ± 30.00 ms (*p* < 0.01, *n* = 455 for the control, *n* = 201 for CCH, *N* = 4; **C**). ^*^Denotes a significant difference between the two distributions. The data are presented as multiple measurements (*n*; dot cloud) recorded for different measurements (*n*) on different hearts (*N*) with the mean ± SEM (solid lines).

The parasympathetic nervous system, on the other hand, is thought to be the dominant branch of the autonomic nervous system. To elicit a parasympathetic response and induce a cholinergic response, 5 μm carbamylcholine was administered to the Goldfish intact heart. As before, the chronotropic properties of the heart were assessed *via* AP and spontaneous heart rate recordings. Not surprisingly, the administration of carbamylcholine altered the AP morphology (Figure 7A) and had a negative chronotropic effect, reducing the heart rate by 92.2% (Figures 7B,C; from 0.98 ± 0.05 Hz to 0.13 ± 0.08 Hz). Not surprisingly, carbamylcholine administration significantly prolonged all three kinetic parameters of the AP (Figures 8A–C; APD30 increased from 235.90 ± 12.10 ms to 295.30 ± 11.50 ms, APD50 increased from 388.10 ± 23.90 ms to 651.40 ± 49.50 ms, and APD90 increased from 446.70 ± 14.60 ms to 833.60 ± 30.00 ms, respectively).

### Parasympathetic Prevalence in Electrocardiographic Signals

**Figure 9:**
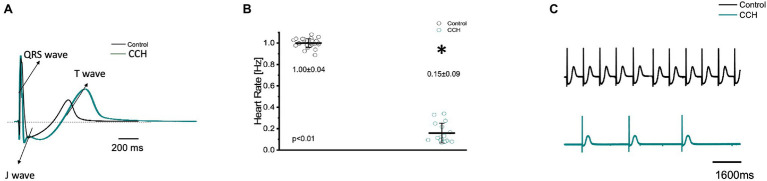
Transmural ventricular electrocardiogram recordings in the absence (black) and presence (green) of 5 μm carbamylcholine. The three main components of the Goldfish electrocardiogram are presented: QRS complex, J wave, and T wave **(A)**. Carbamylcholine administration significantly altered the kinetic parameters of all the electrocardiographic signals. Administration of carbamylcholine significantly decreased heart rate from 1.00 ± 0.04 Hz to 0.15 ± 0.09 Hz (*p* < 0.01, *n* = 29 for the control, *n* = 16 for CCH, *N* = 6; **B**). Electrocardiogram recordings before and after carbamylcholine perfusion reflect a decreased heart rate in response to carbamylcholine **(C)**. ^*^Denotes a significant difference between the two distributions. The data are presented as multiple measurements (*n*; dot cloud) recorded for different measurements (*n*) on different hearts (*N*) with the mean ± SEM (solid lines).

To further assess how cholinergic stimulation altered whole heart electrical activity in the Goldfish model, transmural electrocardiograms were recorded in the presence and absence of 5 μm carbamylcholine (Figure 9). The morphology of the Goldfish electrocardiogram changed dramatically in response to cholinergic stimulation (Figure 9A) and significantly decreased the spontaneous heart rate (Figures 9B,C; from 1.00 ± 0.04 Hz to 0.15 ± 0.09 Hz).

**Figure 10:**
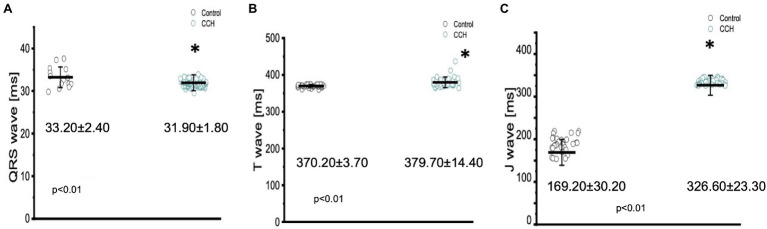
Perfusion with 5 μm carbamylcholine significantly altered the time course of all three components in the Goldfish electrocardiogram. The duration of the QRS significantly decreased from 33.20 ± 2.40 ms to 31.90 ± 1.80 ms (*p* < 0.01, *n* = 15 for the control, *n* = 59 for CCH, *N* = 6; **A**), the T wave increased from 370.20 ± 3.70 ms to 379.70 ± 14.40 ms (*p* < 0.01, *n* = 31 for the control, *n* = 36 for CCH. *N* = 6; **B**), and the J wave show a significant increase from 169.20 ± 30.20 ms to 326.60 ± 23.30 ms (*p* < 0.01, *n* = 32 for the control, *n* = 31 for CCH. *N* = 6; **C**). ^*^Denotes a significant difference between the two distributions. The data are presented as multiple measurements (*n*; dot cloud) recorded for different measurements (*n*) on different hearts (*N*) with the mean ± SEM (solid lines).

Cholinergic stimulation significantly altered the duration of the QRS complex, the T wave, and the J wave (Figure 10). The QRS complex significantly decreased from: 33.20 ± 2.40 ms to 31.90 ± 1.80 ms (Figure 10A), the T wave significantly increased from 370.20 ± 3.70 ms to 379.70 ± 14.40 ms (Figure 10B), and the J wave significantly increased from 169.20 ± 30.20 ms to 326.60 ± 23.30 ms (Figure 10C). The increased J wave duration is likely reflective of the decreased heart rate observed in Figures 7B, 9C.

### Parasympathetic Regulation of Cardiac Contractility

In many vertebrate species, stimulation of either autonomic nervous system branch will not only alter cardiac excitability, but also cardiac contractility. To assess if stimulation of either autonomic nervous system branch altered the inotropic and/or the lusitropic properties of the Goldfish ventricle, experiments were performed in which the amplitude and kinetics of the Ca^2+^ transient were examined in the presence and absence of a cholinergic agonist.

**Figure 11:**
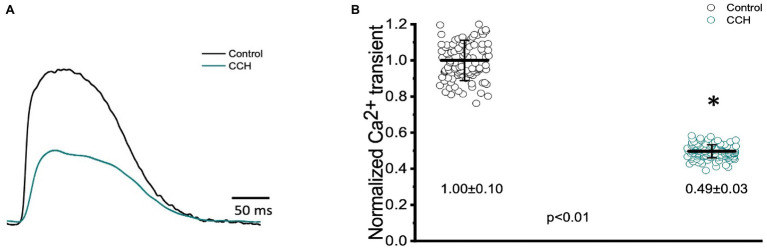
Goldfish ventricular Ca^2+^ transient recording and normalized amplitude in the absence (black) and presence (green) of 5 μm carbamylcholine. Perfusion with carbamylcholine altered the morphology of the Ca^2+^ transient **(A)** and significantly decreased the normalized amplitude of the Ca^2+^ from 1.00 ± 0.10 to 0.49 ± 0.03 (*p* < 0.01, *n* = 108 for the control, *n* = 143 for CCH, *N* = 8; **B**). ^*^Denotes a significant difference between the two distributions. The data are presented as multiple measurements (*n*; dot cloud) recorded for different measurements (*n*) on different hearts (*N*) with the mean ± SEM (solid lines).

To assess cholinergic regulation of contractility specifically, Ca^2+^ transients were recorded from the epicardial wall of the Goldfish ventricle in the presence and absence of 5 μm carbamylcholine (Figure 11). Administration of carbamylcholine had a negative inotropic effect, as the amplitude of the Ca^2+^ transient (Figure 11A) decreased in the presence of carbamylcholine. This negative inotropic effect is also presented in Figure 11B, where the normalized amplitude of the Ca^2+^ transient significantly decreased from 1.00 ± 0.10 to 0.49 ± 0.03 following cholinergic stimulation.

**Figure 12:**
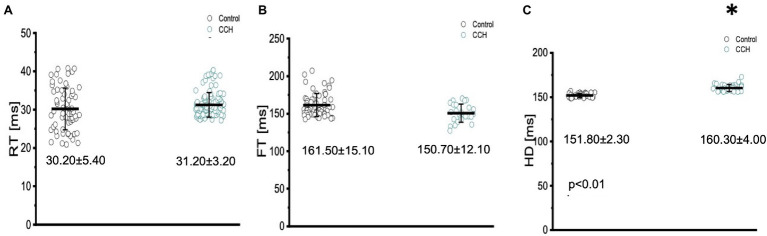
The three kinetical properties of the Ca^2+^ transient, including the rise time (RT), fall time (FT), and half duration (HD) before (black) and after (green) perfusion with 5 μm carbamylcholine. Administration of 5 μm carbamylcholine did not significantly increase the rise time of the Ca^2+^ transient from 30.20 ± 5.40 ms to 31.20 ± 3.20 ms (*p* > 0.01, *n* = 69 for the control, *n* = 85 for CCH, *N* = 8; **A**) or decrease the fall time (FT) from 161.50 ± 15.10 ms to 150.70 ± 12.10 ms (*p* > 0.01, *n* = 59 for the control, *n* = 27 for CCH, *N* = 8; **B**). However, the half duration of the Ca^2+^ transient significantly increased from 151.80 ± 2.30 ms to 160.30 ± 4.00 ms (*p* < 0.01, *n* = 29 for the control, *n* = 31 for CCH, *N* = 8; **C**). ^*^Denotes a significant difference between the two distributions. The data are presented as multiple measurements (*n*; dot cloud) recorded for different measurements (*n*) on different hearts (*N*) with the mean ± SEM (solid lines).

The three kinetical properties of the Ca^2+^ transient, including the rise time (RT), fall time (FT), and half duration (HD) were also evaluated to better understand how stimulation of the cholinergic pathway affected Ca^2+^ handling kinetics in the Goldfish myocardium (Figure 12). Although administration of 5 μm carbamylcholine did not significantly increase the rise time of the Ca^2+^ transient (Figure 12A; 30.20 ± 5.40 ms to 31.20 ± 3.20 ms), it did significantly increase the half duration of the Ca^2+^ transient (Figure 12C; from 151.80 ± 2.30 ms to 160.30 ± 4.00 ms). This effect can be due to the longer APs induced *via* cholinergic stimulation. Interestingly we were unable to observe a significant difference in the relaxation time (Figure 12B; from 161.50 ± 15.10 ms to 150.70 ± 12.10 ms) of the Ca^2+^ transient. This suggests carbamylcholine application did not significantly modify the lusitropic property of the Goldfish myocardium in these experiments; however, a significant change in the half duration of the Ca^2+^ transient does indicate the presence of an intrinsic parasympathetic tone in the Goldfish isolated heart, capable of modifying Ca^2+^ transient kinetics.

## Discussion

In most vertebrate hearts, both excitability and contractility are tightly regulated by the autonomic nervous system. Though there is a significant amount of research regarding the sympathetic and parasympathetic regulation of many vertebrate species, there is little known about how autonomic regulation impacts the electrical and mechanical function of fish hearts specifically. To our knowledge, it is not clear how autonomically driven AP kinetics impact contractility in the intact fish heart, which has become an increasingly popular model used to understand human cardiac physiology and pathophysiology. In this study, we investigated how stimulation of either autonomic branch regulated the time course of APs and electrocardiograms, and how these electrical changes correlated with changes in left ventricular Ca^2+^ transient measurements at the whole heart level. Our results indicate the presence of a fully developed dual control from both the adrenergic and cholinergic nerves in the Goldfish heart, highly resembling the pattern found in other vertebrate models.

### **β**-Adrenergic Stimulation Increased Cardiac Excitability and Contractility

It is well-established stimulation of 𝛽-adrenergic receptors will have a positive chronotropic, dromotropic, inotropic, and lusitropic effect in any vertebrate species exhibiting full autonomic regulation. In the fish model, the autonomous rhythm of the heart is determined by the pacemaker region located near the atrial chamber, identified over 100 years ago (Keith and Mackenzie, 1910). Pacemaker APs are categorized by a gradual and slow diastolic depolarization (Phase 4), toward the threshold voltage of the AP upstroke (Phase 0; Saito, 1973; Harper et al., 1995; Haverinen and Vornanen, 2007; Tessadori et al., 2012). There are three main mechanisms by which an organism can modulate its heart rate, all of which end with an altered slope of the diastolic depolarization during diastole. This slope, set by the sinoatrial node, can be modified positively (by the sympathetic nervous system) or negatively (by the parasympathetic nervous system) by shifting the maximum diastolic potential, or decreasing the rate of depolarization, or (positively or negatively) shifting the membrane potential threshold; all of which could either increase or decrease the time required for the membrane potential to reach the threshold and fire an AP. In the Goldfish model, administration of isoproterenol altered ventricular AP morphology and had a positive chronotropic effect (Figures 1A–C). Remarkably, previous studies have observed isoproterenol to induce strikingly similar AP morphological changes in canine endocardial myocytes and guinea pig cardiomyocytes (O’Hara and Rudy, 2012; Szentandrássy et al., 2012; Sala et al., 2018).

The changes in the AP morphology can be better observed in Figure 2, where the kinetics of the ventricular AP are presented following adrenergic stimulation with isoproterenol. Perfusion with isoproterenol lead to a significant increase in APD30 and APD90 (Figures 2A,C, respectively) and a significant decrease in APD50 (Figure 2B). Interestingly, canine endocardial myocytes treated with isoproterenol exhibit lengthening of APD90 (Szentandrássy et al., 2012; Sala et al., 2018), similar to what we observed for the Goldfish. Unlike mammals, many fish species lack the slow component of the delay rectifier current (IKs), the main current system mediating repolarization effects of adrenergic stimulation on cardiac AP duration (Vornanen, 2017). The absence of this repolarizing current could explain the counterintuitive prolongation of APD30 and APD90 in the presence of an adrenergic stimulus. However, the role of I_Ks_ in response to adrenergic stimulation is not yet elucidated in the Goldfish heart, and further studies are necessary to corroborate this hypothesis.

Transmural electrocardiograms were recorded in the presence and absence of isoproterenol (Figure 3) to examine its effect on whole heart electrical activity. Indeed, isoproterenol perfusion not only altered the morphology of the electrocardiogram (Figure 3A) but also reaffirmed the positive chronotropic effect (Figures 3B,C) of isoproterenol presented in Figure 1. The positive chronotropic effect could be due to an increased slope of diastolic depolarization, as many other mammals exhibit the same pattern in response to adrenergic stimulation (Randall et al., 2020).

Isoproterenol’s significant effect on whole heart excitability (Figure 4) not only reaffirms the presence of a positive chronotropic effect, but also suggests the instigation of a positive dromotropic effect. Because the QRS complex represents ventricular depolarization, its duration indirectly measures intraventricular impulse conduction. Thus, the positive dromotropic effect induced by isoproterenol can best be observed in Figure 4A, where administration of the catecholamine significantly reduced the duration of the QRS complex and increased the rate of intraventricular impulse conduction. Furthermore, isoproterenol significantly increased the duration of the T wave, and significantly prolonged the duration of the J wave (Figures 4B,C). An increased J wave duration is consistent with the prolongation of the APD30 in the presence of 100 nm of isoproterenol (Figure 2A). These modifications observed in the electrocardiogram further solidify the hypothesis that Goldfish exhibit sympathetic regulation, as perfusion with a catecholamine significantly modified cardiac excitability. The cardiac AP alters the mechanical function of vertebrate hearts by increasing intracellular free Ca^2+^ concentration, ultimately inducing cardiac contractions (Coraboeuf, 1978; Randall et al., 2020). In the Goldfish heart, Ca^2+^ influx through the LTCCs is the most likely trigger of Ca^2+^ release from the sarcoplasmic reticulum (Bazmi and Escobar, 2020), which ultimately augments cardiac contractile properties. Therefore, modifications in Ca^2+^ handling dynamics are essential for understanding how cardiac excitability alters cardiac contractility.

Epicardial Ca^2+^transient recordings from the Goldfish ventricle show administration of isoproterenol altered the morphology of the Ca^2+^ transient and significantly increased the normalized amplitude of the Ca^2+^ transient (Figures 5A,B), a trend also observed in guinea pigs (Katra et al., 2004). The positive chronotropic effect (Figures 1C, 3C) in response to adrenergic stimulation could be explained by the alterations present in the Ca^2+^ transient dynamics followed by isoproterenol perfusion. A significant increase in the Ca^2+^ transient amplitude following adrenergic stimulation (Figure 5B) suggests isoproterenol increased the Ca^2+^ current, likely through the LTCC (Bazmi and Escobar, 2020). As previously discussed, adrenergic stimulation activates a cascade of events that phosphorylate numerous Ca^2+^ handling proteins, including PLN on serine 16 and threonine 17. Phosphorylation of PLN removes its inhibitory effect on the cardiac sarcoplasmic endoplasmic reticulum ATPase, thus increasing Ca^2+^ load into the sarcoplasmic reticulum. An increased Ca^2+^ transient amplitude increases the influx of positive charges into the myocardium and reduces the AP threshold; both of which increase the conduction velocity of the AP, resulting in a positive dromotropic effect. Furthermore, an increased Ca^2+^ current will increase the amount of Ca^2+^ in the sarcoplasmic reticulum and ultimately increase Ca^2+^ induced Ca^2+^ release. This would then increase the strength of contraction, resulting in the positive inotropic effect observed in Figure 5. To our knowledge, a positive inotropic effect in response to adrenergic stimulation has yet to be observed in the ventricle of any other fish species (Vornanen and Tuomennoro, 1999; Molina et al., 2007; Vornanen et al., 2010; Abramochkin and Vornanen, 2017). However, it is likely previous studies did not observe positive dromotropic effects considering many of them performed similar experiments on isolated cardiomyocytes and not at the intact heart level. Looking at changes in the kinetic properties of the Goldfish heart, it is likely isoproterenol also had a positive lusitropic effect. Although all three kinetical parameters of the Goldfish Ca^2+^ transient decreased following isoproterenol perfusion (Figures 6A–C), the lusitropic effect can be best observed in Figure 6B, as the fall time of the Ca^2+^ transient significantly decreased, suggesting isoproterenol increased the rate of myocardial relaxation during diastole.

### Muscarinic Stimulation Decreased Cardiac Excitability and Contractility

As previously mentioned, an organism with an adrenergic drive could potentially also have a cholinergic drive, as they are the two antagonistic branches of the autonomic nervous system. Cholinergic control, however, is stronger than adrenergic control and has a negative chronotropic, dromotropic, inotropic, and lusitropic effect (Randall et al., 1968; Urbá-Holmgren et al., 1977; Laurent et al., 1983; Farrell, 1984; Axelsson et al., 1987). In the Goldfish model, perfusing the heart with 5 μm carbamylcholine prolonged the AP (Figure 7A) and had a negative chronotropic effect (Figures 7B,C). The strong negative chronotropic effect induced by cholinergic stimulation could be mediated by an ACh activated potassium current (IK_Ach_); a major current found in fish atrial myocytes responsible for the repolarization of the membrane potential (Molina et al., 2007; Vornanen et al., 2010; Abramochkin and Vornanen, 2017). Furthermore, the activation of a muscarinic receptor will produce inhibition of the adenylyl cyclase reducing the levels of cAMP, preventing PKA-mediated phosphorylation. It is important to note, however, the levels of cAMP are finely regulated by PDEs, which contribute to the lowered cAMP concentrations. Nevertheless, lowered PKA levels result in a reduction in key phosphorylation sites, which ultimately decrease the slope of the diastolic depolarization and decrease heart rate. Furthermore, administration of carbamylcholine significantly prolonged all three kinetical parameters of the AP duration (Figures 8A–C). Considering previous research has shown the presence of Ca^2+^ dependent inactivation of the LTCC in Goldfish ventricular myocytes (Bazmi and Escobar, 2020), it is likely a decreased sarcolemmal Ca^2+^ influx mediated by carbamylcholine (Figure 11B) decelerated inactivation and prolonged the duration of the action potential.

In order to determine how the stimulation of cholinergic response modulated whole heart electrical activity, electrocardiograms were recorded in the presence and absence of carbamylcholine (Figure 9). Perfusion with carbamylcholine altered electrocardiogram morphology (Figure 9A) and reaffirmed the negative chronotropic effect (Figures 9B,C) presented in Figure 7B. Modification of whole heart excitability in response to muscarinic stimulation is presented in Figure 10. Interestingly, the duration of the QRS complex decreased in response to carbamylcholine administration, suggesting a slight positive dromotropic response, something typically observed in tachycardia. Currently, little is known about the depolarizing ventricular currents in the Goldfish which could provide further insight as to why cholinergic stimulation would reduce the duration of the QRS complex. Carbamylcholine perfusion also significantly increased the T and J wave durations (Figures 10A–C); however, the increased J wave was expected as there was a corresponding increase in APD30 (Figure 8A).

The negative chronotropic effect induced by stimulation of the muscarinic receptors could also be explained by modifications of the Ca^2+^ transient. Administration of 5 μM carbamylcholine modified Ca^2+^ transient morphology and significantly decreased the amplitude of the Ca^2+^ transient, suggesting stimulation of muscarinic receptors may have had a negative inotropic effect (Figures 11A,B). This is particularly interesting because previous studies conducted in isolated cardiac myocytes suggest muscarinic stimulation produced minor changes in cardiac chronotropic and inotropic properties in the fish heart (Laurent et al., 1983; Fritsche and Nilsson, 1990; Steele et al., 2009). This discrepancy, however, could be explained by the fact that other experiments were conducted in isolated myocytes, while our experiments were performed in the intact heart. As the heart is an electrically coupled organ, isolation of cardiac myocytes disrupts this electrical coupling, which may alter cardiac contractile properties.

A reduction in the Ca^2+^ current amplitude (Figure 11B) is likely to have reduced the slope of the diastolic depolarization and, as such, induced a negative chronotropic effect. As mentioned before, there are numerous mechanisms by which this slope may change. During cholinergic stimulation, PDEs and inhibition of adenylyl cyclase reduce cAMP levels which not only lower the activation of PKA, but also reduce stimulation of HCN channels. The current produced by these channels, I_f_, typically increases the slope of the diastolic depolarization. However, in the presence of a cholinergic agonist, stimulation of If decreases, thus reducing the slope of the diastolic depolarization and ultimately reducing heart rate. Interestingly, HCN4 pacemaker channels have only been identified in the pacemaker region of the Goldfish and Zebrafish (Tessadori et al., 2012; Newton et al., 2014). Another possible mechanism by which muscarinic receptor stimulation induced a negative chronotropic response could be activation (IK_Ach_), although the contribution of this current is still poorly elucidated in fish ventricular myocytes (Molina et al., 2007; Vornanen et al., 2010; Abramochkin and Vornanen, 2017). Activation of IK_ACh_ would induce hyperpolarization of the maximum diastolic potential, decreasing the heart rate. A decreased Ca^2+^ current and activation of IK_ACh_ also lead to a negative dromotropic effect as a decreased Ca^2+^ current will decrease the influx of positive charges, increase the threshold of the AP, and decrease the mean diastolic potential; all of which reduce AP conduction velocity and induce a negative dromotropic effect.

Modifications presented in the kinetic properties of the Goldfish heart following carbamylcholine perfusion (Figure 12) suggest stimulation of the muscarinic receptor induced a minor negative lusitropic effect. While the half duration of the Ca^2+^ transient significantly increased in response to carbamylcholine perfusion (Figure 12C), the rise time and fall time were not significantly altered (Figures 12A,B). These results are interesting because the effect of carbamylcholine is in the opposite direction of what happens in mouse hearts and is very similar to larger mammals (Aguilar-Sanchez et al., 2019).

## Conclusion

We conclude that the Goldfish heart is a very interesting model to study autonomic regulation due to its similarities with larger mammals. Although the Goldfish heart only has two chambers, its strikingly similar electrophysiological and autonomic characteristics make it a suitable model to study larger mammalian pathophysiology.

## Data Availability Statement

The raw data supporting the conclusions of this article will be made available by the authors, without undue reservation.

## Ethics Statement

The animal study was reviewed and approved by Association for Assessment and Accreditation of Laboratory Animal Care (2008–201).

## Author Contributions

MB and AE designed and performed the research, analyzed data, and wrote the paper. All authors contributed to the article and approved the submitted version.

## Funding

The study was supported by NIH (R01 HL-084487 to AE) and NIH (1R01HL152296 to AE).

## Conflict of Interest

The authors declare the research was conducted in the absence of any commercial or financial relationships that could be construed as a potential conflict of interest.

## Publisher’s Note

All claims expressed in this article are solely those of the authors and do not necessarily represent those of their affiliated organizations, or those of the publisher, the editors and the reviewers. Any product that may be evaluated in this article, or claim that may be made by its manufacturer, is not guaranteed or endorsed by the publisher.

## References

[ref1] Abramochkin D. V. Vornanen M. (2017). Seasonal changes of cholinergic response in the atrium of Arctic navaga cod (Eleginus navaga). J. Comp. Physiol. B. 187, 329–338. doi: 10.1007/s00360-016-1032-y, PMID: 27672043

[ref2] Aguilar-Sanchez Y. Fainstein D. Mejia-Alvarez R. Escobar A. L. (2017). Local field fluorescence microscopy: imaging cellular signals in intact hearts. J. Vis. Exp. 121:55202. doi: 10.3791/55202, PMID: 28362405 PMC5408857

[ref3] Aguilar-Sanchez Y. Rodriguez de Yurre A. Argenziano M. Escobar A. L. Ramos-Franco J. (2019). Transmural autonomic regulation of cardiac contractility at the intact heart level. Front. Physiol. 10:773. doi: 10.3389/fphys.2019.00773, PMID: 31333477 PMC6616252

[ref4] Axelsson M. Ehrenström F. Nilsson S. (1987). Cholinergic and adrenergic influence on the teleost heart *in vivo*. Exp. Biol. 46, 179–186. PMID: 3582588

[ref5] Bazmi M. Escobar A. L. (2020). Excitation–contraction coupling in the goldfish (Carassius auratus) intact heart. Front. Physiol. 11:1103. doi: 10.3389/fphys.2020.01103, PMID: 33041845 PMC7518121

[ref6] Brum G. Osterrieder W. Trautwein W. (1984). Beta-adrenergic increase in the calcium conductance of cardiac myocytes studied with the patch clamp. Eur. J. Phys. 401, 111–118. doi: 10.1007/BF00583870, PMID: 6089094

[ref7] Cohn J. N. (1989). Sympathetic nervous system activity and the heart. Am. J. Hypertens. 2, 353S–356S. PMID: 2532019

[ref8] Collins J. H. Kranias E. G. Reeves A. S. Bilezikjian L. M. Schwartz A. (1981). Isolation of phospholamban and a second proteolipid component from canine cardiac sarcoplasmic reticulum. Biochem. Biophys. Res. Commun. 99, 796–803. doi: 10.1016/0006-291X(81)91235-3, PMID: 6454414

[ref9] Coraboeuf E. (1978). Ionic basis of electrical activity in cardiac tissues. Am. J. Phys. Heart Circ. Phys. 234, H101–H116. doi: 10.1152/ajpheart.1978.234.2.H101, PMID: 341725

[ref10] Escobar A. L. Fernández-Gómez R. Peter J.-C. Mobini R. Hoebeke J. Mijares A. (2006). IgGs and Mabs against the β2-adrenoreceptor block A-V conduction in mouse hearts: a possible role in the pathogenesis of ventricular arrhythmias. J. Mol. Cell. Cardiol. 40, 829–837. doi: 10.1016/j.yjmcc.2006.03.430, PMID: 16697002

[ref11] Escobar A. L. Ribeiro-Costa R. Villalba-Galea C. Zoghbi M. E. Pérez C. G. Mejía-Alvarez R. (2004). Developmental changes of intracellular Ca^2+^ transients in beating rat hearts. Am. J. Phys. Heart Circ. Phys. 286, H971–H978. doi: 10.1152/ajpheart.00308.2003, PMID: 14644760

[ref12] Evans D. B. (1986). Modulation of cAMP: mechanism for positive inotropic action. J. Cardiovasc. Pharmacol. 8(Suppl. 9), S22–S29. doi: 10.1097/00005344-198611001-00003 2433539

[ref13] Farrell A. P. (1984). A review of cardiac performance in the teleost heart: intrinsic and humoral regulation. Can. J. Zool. 62, 523–536. doi: 10.1139/z84-079

[ref14] Ferreiro M. Petrosky A. D. Escobar A. L. (2012). Intracellular Ca^2+^ release underlies the development of phase 2 in mouse ventricular action potentials. Am. J. Phys. Heart Circ. Phys. 302, H1160–H1172. doi: 10.1152/ajpheart.00524.2011, PMID: 22198177 PMC3311451

[ref15] Fritsche R. Nilsson S. (1990). Autonomic nervous control of blood pressure and heart rate during hypoxia in the cod, Gadus morhua. J. Comp. Physiol. B. 160, 287–292. doi: 10.1007/BF00302594

[ref16] Harper A. A. Newton I. P. Watt P. W. (1995). The effect of temperature on spontaneous action potential discharge of the isolated sinus venosus from winter and summer plaice (Pleuronectes platessa). J. Exp. Biol. 198, 137–140. doi: 10.1242/jeb.198.1.137 9317498

[ref17] Haverinen J. Vornanen M. (2007). Temperature acclimation modifies sinoatrial pacemaker mechanism of the rainbow trout heart. Am. J. Phys. Regul. Integr. Comp. Phys. 292, R1023–R1032. doi: 10.1152/ajpregu.00432.2006, PMID: 17008459

[ref18] Hayes J. S. Mayer S. E. (1981). Regulation of Guinea pig heart phosphorylase kinase by cAMP, protein kinase, and calcium. Am. J. Physiol. Endocrinol. Metab. 240, E340–E349. doi: 10.1152/ajpendo.1981.240.3.E340, PMID: 6259950

[ref19] Henning R. J. (1992). Vagal stimulation during muscarinic and β-adrenergic blockade increases atrial contractility and heart rate. J. Auton. Nerv. Syst. 40, 121–129. doi: 10.1016/0165-1838(92)90023-A, PMID: 1464693

[ref20] Hildebrandt J. D. Sekura R. D. Codina J. Iyengar R. Manclark C. R. Birnbaumer L. (1983). Stimulation and inhibition of adenylyl cyclases mediated by distinct regulatory proteins. Nature 302, 706–709. doi: 10.1038/302706a0, PMID: 6300694

[ref21] Katra R. P. Pruvot E. Laurita K. R. (2004). Intracellular calcium handling heterogeneities in intact Guinea pig hearts. Am. J. Phys. Heart Circ. Phys. 286, H648–H656. doi: 10.1152/ajpheart.00374.2003, PMID: 14551057

[ref22] Katz A. M. (1967). Regulation of cardiac muscle contractility. J. Gen. Physiol. 50, 185–196. doi: 10.1085/jgp.50.6.185, PMID: 4227923 PMC2225748

[ref23] Keith A. Mackenzie I. (1910). Recent researches on the anatomy of the heart. Lancet 175, 101–103. doi: 10.1016/S0140-6736(01)74711-3

[ref24] Kornyeyev D. Reyes M. Escobar A. L. (2010). Luminal Ca^2+^ content regulates intracellular Ca^2+^ release in subepicardial myocytes of intact beating mouse hearts: effect of exogenous buffers. Am. J. Phys. Heart Circ. Phys. 298, H2138–H2153. doi: 10.1152/ajpheart.00885.2009, PMID: 20382849 PMC2886618

[ref25] Krebs E. G. (1972). Protein kinases. Curr. Top. Cell. Regul. 5, 99–133. doi: 10.1016/B978-0-12-152805-8.50010-1 4358204

[ref26] Laurent P. Holmgren S. Nilsson S. (1983). Nervous and humoral control of the fish heart: structure and function. Comp. Biochem. Physiol. A Physiol. 76, 525–542. doi: 10.1016/0300-9629(83)90455-3

[ref27] Lee W. C. Shideman F. E. (1959). Role of myocardial Catecholamines in cardiac contractility. Science 129, 967–968. doi: 10.1126/science.129.3354.967 13646629

[ref28] Lindemann J. P. Watanabe A. M. (1985). Muscarinic cholinergic inhibition of beta-adrenergic stimulation of phospholamban phosphorylation and Ca^2+^ transport in Guinea pig ventricles. J. Biol. Chem. 260, 13122–13129. doi: 10.1016/S0021-9258(17)38847-6, PMID: 2414274

[ref29] López Alarcón M. M. Rodríguez de Yurre A. Felice J. I. Medei E. Escobar A. L. (2019). Phase 1 repolarization rate defines Ca^2+^ dynamics and contractility on intact mouse hearts. J. Gen. Physiol. 151, 771–785. doi: 10.1085/jgp.201812269, PMID: 31000581 PMC6571993

[ref30] Marks A. R. (2013). Calcium cycling proteins and heart failure: mechanisms and therapeutics. J. Clin. Investig. 123, 46–52. doi: 10.1172/JCI62834, PMID: 23281409 PMC3533269

[ref31] Mattiazzi A. Argenziano M. Aguilar-Sanchez Y. Mazzocchi G. Escobar A. L. (2015). Ca2+ Sparks and Ca2+ waves are the subcellular events underlying Ca2+ overload during ischemia and reperfusion in perfused intact hearts. J. Mol. Cell. Cardiol. 79, 69–78. doi: 10.1016/j.yjmcc.2014.10.011, PMID: 25451173 PMC4302011

[ref32] Mejía-Alvarez R. Manno C. Villalba-Galea C. A. del Valle Fernández L. Costa R. Fill M.. (2003). Pulsed local-field fluorescence microscopy: A new approach for measuring cellular signals in the beating heart. Pflugers Arch. 445, 747–758. doi: 10.1007/s00424-002-0963-1, PMID: 12632197

[ref33] Mersereau E. J. Poitra S. L. Espinoza A. Crossley D. A. Darland T. (2015). The effects of cocaine on heart rate and electrocardiogram in zebrafish (Danio rerio). Comp. Bioche. Physiol. C Toxicol. Pharmacol. 172-173, 1–6. doi: 10.1016/j.cbpc.2015.03.007, PMID: 25847362 PMC4458413

[ref34] Molina C. E. Gesser H. Llach A. Tort L. Hove-Madsen L. (2007). Modulation of membrane potential by an acetylcholine-activated potassium current in trout atrial myocytes. Am. J. Phys. Regul. Integr. Comp. Phys. 292, R388–R395. doi: 10.1152/ajpregu.00499.2005, PMID: 16959867

[ref35] Nakamura T. Lozano P. R. Ikeda Y. Iwanaga Y. Hinek A. Minamisawa S.. (2002). Fibulin-5/DANCE is essential for elastogenesis *in vivo*. Nature 415, 171–175. doi: 10.1038/415171a, PMID: 11805835

[ref36] Newton C. M. Stoyek M. R. Croll R. P. Smith F. M. (2014). Regional innervation of the heart in the goldfish, Carassius auratus: a confocal microscopy study: innervation of the goldfish heart. J. Comp. Neurol. 522, 456–478. doi: 10.1002/cne.23421, PMID: 23853005

[ref37] O’Hara T. Rudy Y. (2012). Quantitative comparison of cardiac ventricular myocyte electrophysiology and response to drugs in human and nonhuman species. Am. J. Phys. Heart Circ. Phys. 302, H1023–H1030. doi: 10.1152/ajpheart.00785.2011, PMID: 22159993 PMC3311457

[ref38] Osterrieder W. Brum G. Hescheler J. Trautwein W. Flockerzi V. Hofmann F. (1982). Injection of subunits of cyclic AMP-dependent protein kinase into cardiac myocytes modulates Ca2+ current. Nature 298, 576–578. doi: 10.1038/298576a0, PMID: 6285199

[ref39] Randall W. C. Randall D. C. Ardell J. L. (2020). “Autonomic regulation of myocardial contractility,” in Reflex Control of the Circulation. 1st *Edn*. eds. Zucker I. H. Gilmore J. P. (United Kingdom: Taylor and Francis), 67–103.

[ref40] Randall W. Wechsler J. Pace J. Szentivanyi M. (1968). Alterations in myocardial contractility during stimulation of the cardiac nerves. Am. J. Physiol. 214, 1205–1212. doi: 10.1152/ajplegacy.1968.214.5.1205, PMID: 5647196

[ref41] Saito T. (1973). Effects of vagal stimulation on the pacemaker action potentials of carp heart. Comp. Biochem. Physiol. A Physiol. 44, 191–199. doi: 10.1016/0300-9629(73)90381-2, PMID: 4404863

[ref42] Sala L. Hegyi B. Bartolucci C. Altomare C. Rocchetti M. Váczi K.. (2018). Action potential contour contributes to species differences in repolarization response to β-adrenergic stimulation. Europace 20, 1543–1552. doi: 10.1093/europace/eux236, PMID: 29045640

[ref43] Sandblom E. Axelsson M. (2011). Autonomic control of circulation in fish: A comparative view. Auton. Neurosci. 165, 127–139. doi: 10.1016/j.autneu.2011.08.006, PMID: 21925970

[ref44] Steele S. L. Lo K. H. A. Li V. W. T. Cheng S. H. Ekker M. Perry S. F. (2009). Loss of M_2_ muscarinic receptor function inhibits development of hypoxic bradycardia and alters cardiac β-adrenergic sensitivity in larval zebrafish (*Danio rerio*). Am. J. Phys. Regul. Integr. Comp. Phys. 297, R412–R420. doi: 10.1152/ajpregu.00036.2009, PMID: 19515979

[ref45] Suko J. Maurer-Fogy I. Plank B. Bertel O. Wyskovsky W. Hohenegger M.. (1993). Phosphorylation of serine 2843 in ryanodine receptor-calcium release channel of skeletal muscle by cAMP-, cGMP- and CaM-dependent protein kinase. Biochim. Biophys. Acta 1175, 193–206. doi: 10.1016/0167-4889(93)90023-I, PMID: 8380342

[ref46] Szentandrássy N. Farkas V. Bárándi L. Hegyi B. Ruzsnavszky F. Horváth B.. (2012). Role of action potential configuration and the contribution of Ca^2+^ and K^+^ currents to isoprenaline-induced changes in canine ventricular cells: isoprenaline in canine heart. Br. J. Pharmacol. 167, 599–611. doi: 10.1111/j.1476-5381.2012.02015.x, PMID: 22563726 PMC3449264

[ref47] Tessadori F. van Weerd J. H. Burkhard S. B. Verkerk A. O. de Pater E. Boukens B. J.. (2012). Identification and functional characterization of cardiac pacemaker cells in Zebrafish. PLoS One 7:e47644. doi: 10.1371/journal.pone.0047644, PMID: 23077655 PMC3473062

[ref48] Tsai C.-T. Wu C.-K. Chiang F.-T. Tseng C.-D. Lee J.-K. Yu C.-C.. (2011). *In-vitro* recording of adult zebrafish heart electrocardiogram—A platform for pharmacological testing. Clin. Chim. Acta 412, 1963–1967. doi: 10.1016/j.cca.2011.07.002, PMID: 21767531

[ref49] Urbá-Holmgren R. González R. M. Holmgren B. (1977). Is yawning a cholinergic response? Nature 267, 261–262. doi: 10.1038/267261a0 865617

[ref50] Valdivia H. H. Kaplan J. H. Ellis-Davies G. C. Lederer W. J. (1995). Rapid adaptation of cardiac ryanodine receptors: modulation by Mg^2+^ and phosphorylation. Science 267, 1997–2000. doi: 10.1126/science.7701323, PMID: 7701323 PMC4242209

[ref51] Valverde C. A. Kornyeyev D. Ferreiro M. Petrosky A. D. Mattiazzi A. Escobar A. L. (2010). Transient Ca2+ depletion of the sarcoplasmic reticulum at the onset of reperfusion. Cardiovasc. Res. 85, 671–680. doi: 10.1093/cvr/cvp371, PMID: 19920131 PMC2819836

[ref52] Valverde C. A. Mundiña-Weilenmann C. Reyes M. Kranias E. G. Escobar A. L. Mattiazzi A. (2006). Phospholamban phosphorylation sites enhance the recovery of intracellular Ca^2+^ after perfusion arrest in isolated, perfused mouse heart. Cardiovasc. Res. 70, 335–345. doi: 10.1016/j.cardiores.2006.01.018, PMID: 16516179

[ref53] Vornanen M. (2017). “Electrical excitability of the fish heart and its autonomic regulation,” in Fish Physiology. *Vol*. 36. eds. Gamperl A. K. Gillis T. E. Farrell A. P. Brauner C. J. (New York: Elsevier), 99–153.

[ref54] Vornanen M. Hälinen M. Haverinen J. (2010). Sinoatrial tissue of crucian carp heart has only negative contractile responses to autonomic agonists. BMC Physiol. 10:10. doi: 10.1186/1472-6793-10-10, PMID: 20540719 PMC2894799

[ref55] Vornanen M. Tuomennoro J. (1999). Effects of acute anoxia on heart function in crucian carp: importance of cholinergic and purinergic control. Am. J. Phys. 277, R465–R475. doi: 10.1152/ajpregu.1999.277.2.R465, PMID: 10444553

[ref56] Watanabe A. M. Lindemann J. P. (1984). “Mechanisms of adrenergic and cholinergic regulation of myocardial contractility,” in Physiology and Pathophysiology of the Heart. *Vol*. 34. ed. Sperelakis N. (United States: Springer), 377–404.

[ref57] Weilenmann C. M. Vittone L. Cingolani G. Mattiazzi A. (1987). Dissociation between contraction and relaxation: the possible role of phospholamban phosphorylation. Basic Res. Cardiol. 82, 507–516. doi: 10.1007/BF01907220, PMID: 2963614

[ref58] Xing N. Ji L. Song J. Ma J. Li S. Ren Z.. (2017). Cadmium stress assessment based on the electrocardiogram characteristics of zebra fish (Danio rerio): QRS complex could play an important role. Aquat. Toxicol. 191, 236–244. doi: 10.1016/j.aquatox.2017.08.015, PMID: 28869925

